# Low-flux scanning electron diffraction reveals substructures inside the ordered membrane domain

**DOI:** 10.1038/s41598-020-79083-7

**Published:** 2020-12-21

**Authors:** Masanao Kinoshita, Shimpei Yamaguchi, Nobuaki Matsumori

**Affiliations:** Department of Chemistry, Graduate School of Science, Kyushu University, Fukuoka, 819-0395 Japan

**Keywords:** Membrane biophysics, Membrane structure and assembly

## Abstract

Ordered/disordered phase separation occurring in bio-membranes has piqued researchers’ interest because these ordered domains, called lipid rafts, regulate important biological functions. The structure of the ordered domain has been examined with artificial membranes, which undergo macroscopic ordered/disordered phase separation. However, owing to technical difficulties, the local structure inside ordered domains remains unknown. In this study, we employed electron diffraction to examine the packing structure of the lipid carbon chains in the ordered domain. First, we prepared dehydrated monolayer samples using a rapid-freezing and sublimation protocol, which attenuates the shrinkage of the chain-packing lattice in the dehydration process. Then, we optimised the electron flux to minimise beam damage to the monolayer sample. Finally, we developed low-flux scanning electron diffraction and assessed the chain packing structure inside the ordered domain formed in a distearoylphosphatidylcholine/dioleoylphosphatidylcholine binary monolayer. Consequently, we discovered that the ordered domain contains multiple subdomains with different crystallographic axes. Moreover, the size of the subdomain is larger in the domain centre than that near the phase boundary. To our knowledge, this is the first study to reveal the chain packing structures inside an ordered domain.

## Introduction

Cell membranes, which consist of a large variety of phospholipids, show lateral heterogeneity in terms of lipid distribution. Such heterogeneous distribution leads to ordered/disordered phase separation. Since the late twentieth century, researchers have been interested in the ordered membrane domains due to their potential involvement in important biological functions^1^. For instance, it is widely believed that some membrane proteins are transiently entrapped in ordered domains, facilitating transmembrane signalling. Despite their hypothesised importance in membrane biology, detailed information on ordered domains is limited, owing to their low spatiotemporal stability^2–4^; the putative size of the domains in quiescent cells is less than 200 nm and their lifetime is less than a few hundred milliseconds.

So far, the physicochemical properties and structure of the ordered domains have been examined with artificial mono- and bi-layer membranes. For example, binary mixtures of saturated phospholipids or sphingomyelins (SMs) with unsaturated phospholipids undergo phase segregation between the ordered and disordered membrane domains^5,6^. In the case of bilayer systems, the addition of cholesterol (chol) to the binary mixtures forms another type of ordered domain called liquid ordered (Lo) phase^7–11^. Because these ordered domains are more stable and significantly larger than those formed in bio-membranes, artificial membranes are useful to obtain basic information on ordered domains such as geometry^12,13^, lipid composition^7,14,15^, and thermal stability^16^. Recently, some studies have indicated that ordered domains are not homogeneous but consist of multiple subdomains. For example, a high-speed single-particle tracking experiment demonstrated that a gold nanoparticle labelled dipalmitoyl-phosphatidylethanolamine (GNP-DPPE) does not have simple Brownian motion in ordered domains, which are formed in dipalmitoylphosphatidylcholine (DPPC)/diphytanoylphosphatidylcholine/cholesterol (chol) ternary supported bilayers^17^. They speculated that GNP-DPPE is entrapped in the subdomains, hampering its normal diffusion in the ordered domain. Moreover, fluorescent lifetime measurements^18^ and Raman spectroscopy^19^ revealed that SMs forms small aggregations in SM/dioleoylphosphatidylcholine (DOPC) binary bilayers, while fluorescent microscopy identified the macroscopic ordered/disordered phase separation in these mixtures^5^. These facts indicate that aggregations of SMs exist in macroscopic ordered domains^18^. Although there are several lines of evidence for the existence of subdomains^20^, the techniques used could not address lipid packing structures inside the ordered membrane domain.

Wide angle X-ray diffraction (WAXD) has been used for the investigation of lipid packing structures in artificial membranes^21–24^ because the WAXD shows diffraction peaks corresponding to lattice spacings of the lipid carbon chains. However, the diameter of the X-rays frequently used for those experiments is more than 100 μm, which is much larger than the size of the ordered domain. Consequently, a WAXD pattern contains integrated information on a number of ordered and disordered domains residing within the beam size. In addition, owing to the limitation of the scattering efficiency of the X-rays, the WAXD peaks from a single membrane domain are too weak to obtain reliable results. Therefore, another method is needed to examine the local structures inside a single ordered domain.

Instead of WAXD, electron diffraction (ED) is a promising method for local-structure analysis due to the following reasons: First, because ED is performed with an electron microscope, we can direct the electron beam to a micro-size target under visual observation. Second, using a selector aperture, we can select the ED patterns only from the desired regions, whose size is less than 1 µm in diameter. Additionally, due to its significantly greater diffraction potency than X-rays, ED likely provides strong diffraction peaks even from a single membrane domain. While ED experiments were sometimes used for structural analysis of lipid membranes from the 1970s to the 1990s^25−32^, the techniques progression has been hampered by some technical difficulties. For example, ED requires a dry and fixed sample because it is performed under a high vacuum. In addition, compared with X-rays, the electron beam causes significant damage to the lipid membranes^33,34^.

In the present study, we prepare dehydrated samples employing a rapid-freezing and sublimation (RFS) protocol, hampering the shrinkage of the chain-packing lattice in the dehydration process. In addition, we optimised electron flux to minimise beam damage to the lipid membranes. Finally, we revealed the local structures inside a single ordered domain by newly developed low-flux scanning electron diffraction (LFSED).

## Results

### Influence of the rapid-freezing and sublimation (RFS) on the chain packing in lipid monolayer

It has previously been reported that dehydration of lipid membranes causes shrinkage of the chain packing lattice effectively in the disordered membranes^25^. To overcome this problem, we employed the RFS method to prepare dehydrated samples (see “Materials and methods” for details). Because lipid diffusion is fixed through the RFS protocol, it should create dehydrated samples without the shrinkage of the chain-packing lattice.

**Figure 1:**
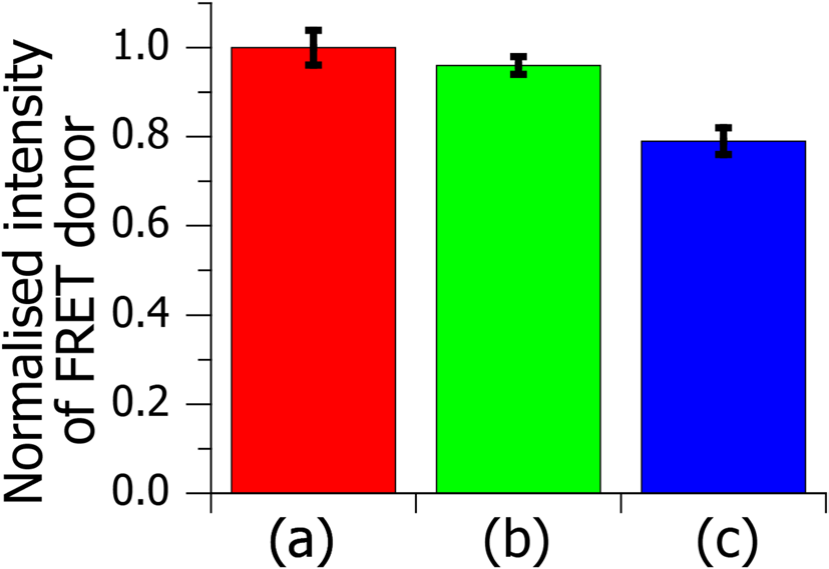
FRET-quenching intensity of the donor in wet and dehydrated DOPC monolayers. The samples contain 0.2 mol% Bodipy-PC and 0.4 mol% Texas Red labelled DPPE (Texas Red-DPPE) as FRET donor and acceptor, respectively. Bars show the donor intensity *I*_donor_ of (**a**) a wet sample, (**b**) a sample dehydrated by the RFS method and (**c**) a sample dehydrated at the atmospheric pressure and room temperature. For easy comparison, the *I*_donor_-values of the dehydrated samples were normalised to that of the wet sample. Error bars indicate the standard errors.

This could be confirmed by fluorescent energy transfer (FRET) measurements. Since FRET sensitively depends on the distance between the FRET donor and acceptor in membranes, shrinkage of the chain-packing lattice causes effective FRET-quenching of the donor fluorescence. Figure 1 shows normalised donor intensity *I*_donor_ in DOPC supported monolayers, which form a homogeneous disordered membrane on the air–water interface^35^. The dehydration of the sample at atmospheric pressure and room temperature caused effective FRET-quenching of the donor intensity (compare Fig. 1a and c), indicating that the packing shrinkage occurs in the dehydration process. On the other hand, the *I*_donor_-value of the RFS sample is consistent with that of the wet sample within the error range (compare Fig. 1a and b). While the RFS method has been widely used for electron microscopy and mass-based imaging^36−38^, we demonstrated for the first time that it does not cause significant shrinkage of the chain packing lattice in the disordered monolayer.

### Kinetics of structural decay caused by electron beam irradiation

We optimised the flux of the electron beam to minimise beam damage to the sample. Here, we used a distearoylphosphatidylcholine (DSPC) monolayer, which forms an ordered phase at air–water interface^39^. Because the DSPC monolayer provides a clear reflection, it is convenient for optimisation. We set the wavelength and image acquisition time to be 0.0037 nm (acceleration voltage *V*_acc_ = 100 keV) and five s, respectively. The sample was kept at − 180 °C with a cooling holder and the beam damage was reduced.

**Figure 2:**
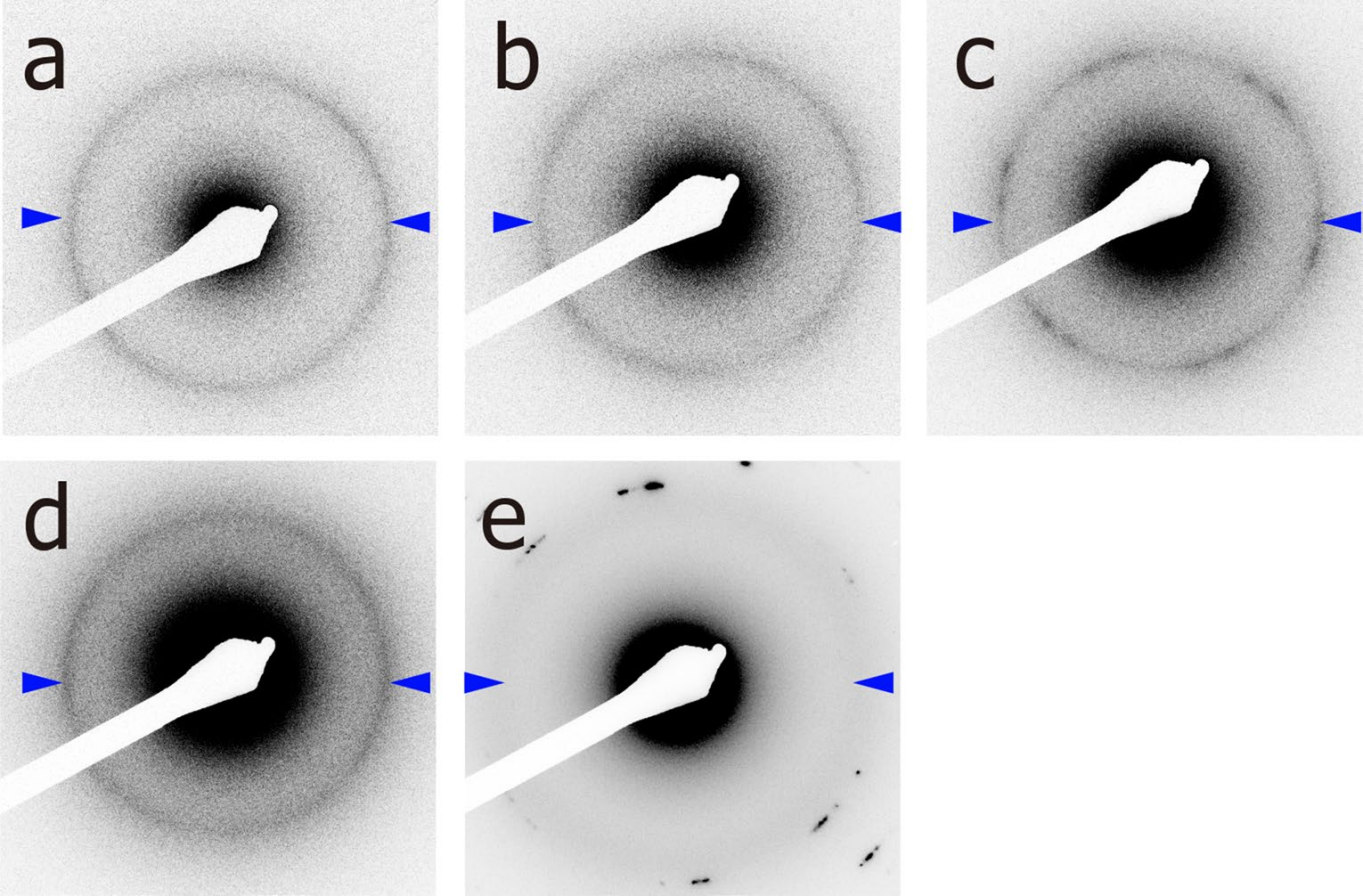
ED patterns from the DSPC monolayer at different electron fluxes; (**a**) 0.9 e/nm^2^·s, (**b**) 2.3 e/nm^2^·s, (**c**) 4.7 e/nm^2^·s, (**d**) 15.9 e/nm^2^·s, and (**e**) 30.0 e/nm^2^·s. The DSPC monolayer was formed on a collodion-coated grid for transmission electron microscopic observation (TEM-grid). The selected area was 7.9 μm^2^, corresponding to 1 μm in diameter, and the exposure time was five s. Arrowheads indicate diffraction peaks corresponding to the carbon-chain packing. To improve the visibility, we have shown the black/white-inverse images.

**Figure 3:**
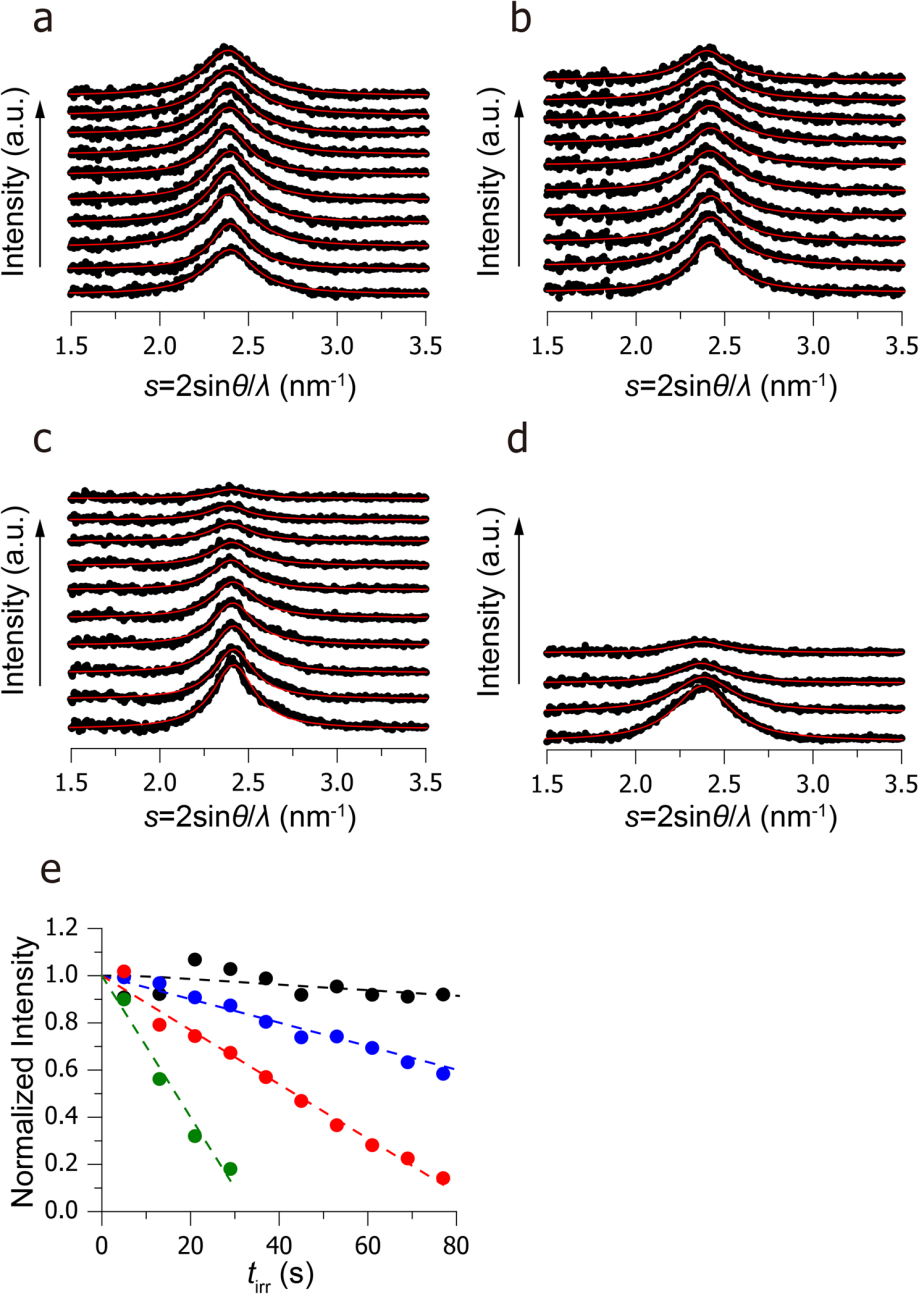
Kinetics of structural decay upon continuous irradiation with an electron beam. One-dimensional ED patterns at the electron flux of (**a**) 0.9 e/nm^2^·s, (**b**) 2.3 e/nm^2^·s, (**c**) 4.7 e/nm^2^·s, and (**d**) 15.9 e/nm^2^·s are shown. The data were obtained in the same region under continuous irradiation with the electron beam. The irradiation times *t*_irr_ were 5 s, 13 s, 21 s, 29 s, 37 s, 45 s, 53 s, 61 s, 69 s, and 77 s from the bottom to top profiles. Each profile is fitted to a Lorentz function, and the fitting result is shown by a red profile. The selected area is 0.79 μm^2^, which corresponds to 1 μm in diameter. (**e**) Peak heights are plotted as a function of *t*_irr_ under the electron flux of 0.9 e/nm^2^·s (black), 2.3 e/nm^2^·s (blue), 4.7 e/nm^2^·s (red), and 15.9 e/nm^2^·s (green). The dashed lines indicate linear fitting in the region *t*_irr_ < 77 s for 0.9 e/nm^2^·s − 4.7 e/nm^2^·s and *t*_irr_ < 29 s for 15.9 e/nm^2^·s.

**Figure 4:**
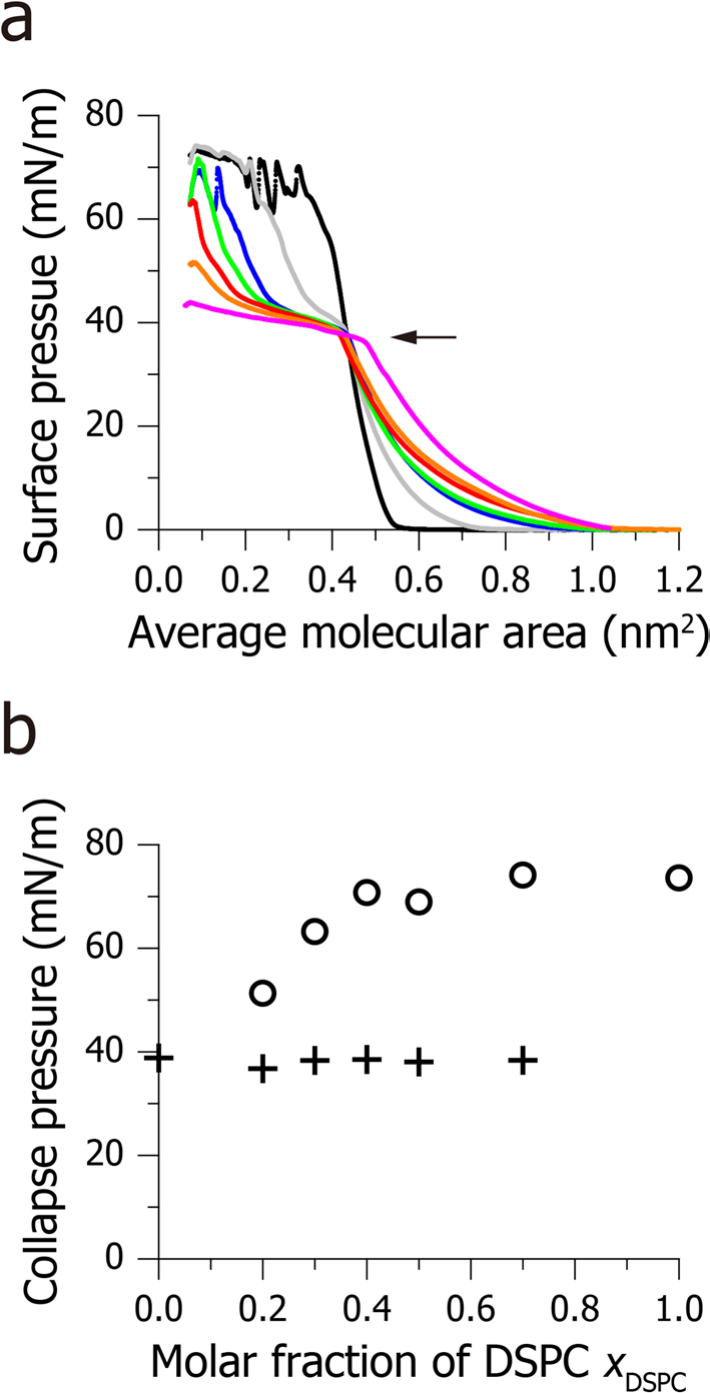
Composition-dependent phase behaviour of DSPC/DOPC binary monolayers. (**a**) *π*–*A* isotherms of DSPC/DOPC binary monolayers at different mole fractions of DSPC (*x*_DSPC_). *x*_DSPC_ = 0 (pink), 0.2 (orange), 0.3 (red), 0.4 (green), 0.5 (blue), 0.7 (grey), and 1.0 (black). An arrow indicated the collapse pressure of the disordered domains. (**b**) Collapse pressure vs. composition plot. The circles and crosses show the collapse pressure of the ordered and disordered domains, respectively.

Figure 2 shows the diffraction patterns from a DSPC monolayer at different electron fluxes. The irradiation at 30.0 e/nm^2^·s gave rise to a broad ring pattern (indicated by arrowheads in Fig. 2e), indicating immediate disruption of the chain-packing structure upon such strong irradiation. Some strong spots appearing in the wide angular regions are unlikely to have originated from the carbon chains because their peak positions (> 3.0 nm^−1^; Supplementary Fig. S1) are far from the lattice spacing of the chain packing. Next, we reduced the electron flux to less than 15.9 e/nm^2^·s and successfully obtained a sharp Debye–Scherrer diffraction at 2.40 ± 0.01 nm^−1^ (indicated by arrowheads in Fig. 2a–d and their one-dimensional profiles are shown in the bottom of Fig. 3a–d). By applying the lattice spacing *d* = 0.42 nm (= 1/2.40 nm) to Eq. (2) that is described in “Materials and methods”, we calculated the lateral molecular area of the DSPC to be 0.41 nm^2^. This value is close to the molecular area of the DSPC on the water subphase obtained in the *π–A* isotherm measurement (0.45 nm^2^ at *π* = 30 mN/m; a black isotherm in Fig. 4a). Also, this result is likely reasonable because it was previously reported that that the chain-packing structure of an ordered membrane (the gel phase) is hardly affected by dehydration^25^.

Figure 3a–d shows one-dimensional ED profiles upon continuous irradiation with electron beams, demonstrating that the peak intensity decreases as the irradiation duration *t*_irr_ increases. To quantitatively analyse the structural decay, we plotted the normalised intensity *I*_norm_ as a function of *t*_irr_ (Fig. 3e). Fitting these data to a linear function (dashed lines in Fig. 3e) revealed that the peak intensity decreases in the initial five seconds by 0.4 ± 5%, 2.8 ± 2%, 5.8 ± 4%, and 15.0 ± 7% at electron fluxes of 0.9 e/nm^2^·s, 2.3 e/nm^2^·s, 4.7 e/nm^2^·s, and 15.9 e/nm^2^·s, respectively. Considering their errors, we concluded that the structural decay is sufficiently small to be considered negligible upon irradiation with an electron flux of less than 4.7 e/nm^2^·s.

Moreover, we examined the effect of the wavelength *λ*_e_ on the structural decay by comparing the red plots in Fig. 3e (*λ*_e_ = 0.0037 nm; *V*_acc_ = 100 keV) and in Supplementary Fig. S2 (*λ*_e_ = 0.0021 nm; *V*_acc_ = 200 keV). Consequently, the decrease in the *I*_norm_-value became moderate at the shorter wavelength. However, such shorter wavelength caused a large deviation in the time curve of the *I*_norm_-value (Supplementary Fig. S2). Probably, our TEM could not supply stable electron beam under the high acceleration voltage (*V*_acc_ = 200 keV) and, thus, the structural decay of the sample did not show a linear correlation with *t*_irr_. Taking account of these results, we used a beam flux of less than 4.7 e/nm^2^·s and a wavelength of 0.0037 nm (*V*_acc_ = 100 keV) in the following ED experiments.

### Macroscopic phase separation in DSPC/DOPC binary monolayers

While there are some previous studies using DSPC/DOPC binary monolayers^39,40^, their phase behaviour remains unknown. Here, we investigated the composition-dependent phase behaviour of the DSPC/DOPC monolayers by surface pressure-molecular area isotherm (*π–A* isotherm) measurements. Figure 4a shows the *π–A* isotherms of monolayers consisting of DSPC, DOPC, and their mixtures. The surface pressure of the pure DSPC and pure DOPC monolayers increased steadily as the monolayers were laterally compressed (black and pink isotherms, respectively, in Fig. 4a). On the other hand, DSPC/DOPC monolayers (0.2 ≤ *x*_DSPC_ ≤ 0.7) show a stepwise increase in surface pressure. This result indicates that the DSPC/DOPC monolayers undergo macroscopic phase separation; namely, the DOPC-rich disordered domains collapse at lower surface pressure (indicated by an arrow in Fig. 4a) and the DSPC-rich ordered domains at higher surface pressure (*π* > 40 mN/m)^5^. Here, the disordered domain is formed by almost pure DOPC because the collapse pressure of the disordered domain (*π* ~ 40 mN/m) is consistent with that of the pure DOPC monolayer across the experimental compositional range (crosses in Fig. 4b). On the other hand, in the region of 0.2 ≤ *x*_DSPC_ < 0.4, the collapse pressure of the ordered domain is significantly smaller than that of pure DSPC (circles in Fig. 4b), indicating that the ordered domain does not consist of pure DSPC, but contains small amounts of DOPC. In the following experiments, we analyse the lipid packing structure of the ordered domain formed in the DSPC/DOPC (*x*_DSPC_ = 0.3) monolayer because the structure of the pure DSPC monolayer has already been obtained in Figs. 2 and 3.

### Chain packing structures inside a single ordered membrane domain

**Figure 5:**
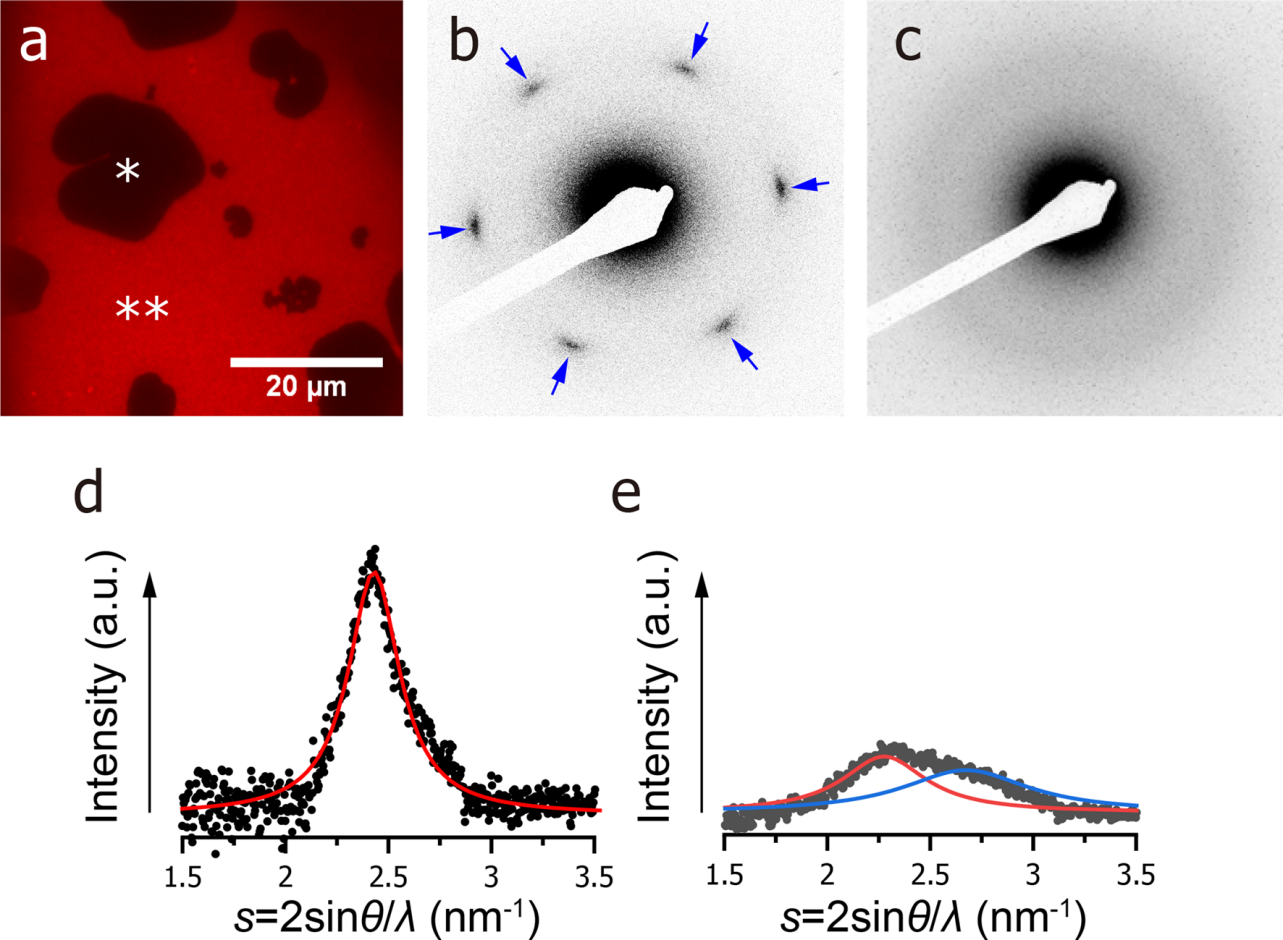
Selective acquisition of ED patterns from the ordered and disordered domains. (**a**) A fluorescent micrograph of the DSPC/DOPC (*x*_DSPC_ = 0.3) monolayer formed on a collodion-coated TEM-grid. Since sample contain 0.2 mol% Texas Red-DPPE, a disordered domain marker, the (*) darker and (**) brighter regions correspond to the ordered and disordered domains, respectively. (**b**) and (**c**) show ED patterns obtained in the ordered and disordered domains, respectively. Arrows indicate the hexagonal spot obtained in the ordered domain. The selected area is 0.79 μm^2^, corresponding to a diameter of 1 μm. An exposure time of five seconds was used. (**d**) and (**e**) show the one-dimensional ED profiles of (**b**) and (**c**), respectively. Although we subtracted the background using a monolayer-free TEM-grid (see “Materials and methods” for details), the diffraction peak from the collodion film could not be completely removed in the panel (**e**). This is because the thickness of the collodion film is not homogeneous on the TEM-grid. Thus, we deconvoluted those peaks using the two Lorentz functions; the red and blue profiles show the diffraction peaks corresponding to the carbon chains and the collodion film, respectively.

Figure 5a shows a fluorescent micrograph of the DSPC/DOPC monolayer, which is formed on the collodion-coated TEM grid. Since this sample contains 0.2 mol% Texas Red-DPPE, a disordered phase marker^41^, the darker and brighter regions correspond to the DSPC-rich ordered and DOPC-rich disordered domains, respectively. In the following experiments, we determined the beam positions, referring to the domain distribution shown in the fluorescent micrographs (see “Materials and methods” and Supplementary Fig. S3 for details).

Figure 5b shows the ED patterns from the 0.79 μm^2^ region inside the ordered domain, giving rise to sharp hexagonal spots. To gain further insight into the chain-packing structure, we represented the one-dimensional intensity of the hexagonal spots and found that the ordered domain gives a single sharp peak at 2.40 nm^−1^ (Fig. 5d). These results revealed that the carbon chains aligned perpendicular to the monolayer surface with a lattice spacing of 0.42 (= 1/2.40) nm. On the other hand, the disordered domain, which consists of almost pure DOPC, gave a broad Debye–Scherrer pattern centred at 2.26 nm^−1^ (Fig. 5c and a red profile in Fig. 5e). This ED pattern is similar to the WAXD pattern of the disordered DOPC bilayers^42^; DOPC bilayers also give a broad WAXD peak and the peak position is roughly estimated to be 2.23 nm^−1^. This fact indicates that the RFS protocol does not cause significant shrinkage of the chain-packing lattice in the disordered monolayer, being in line with FRET experiments (Fig. 1).

**Figure 6:**
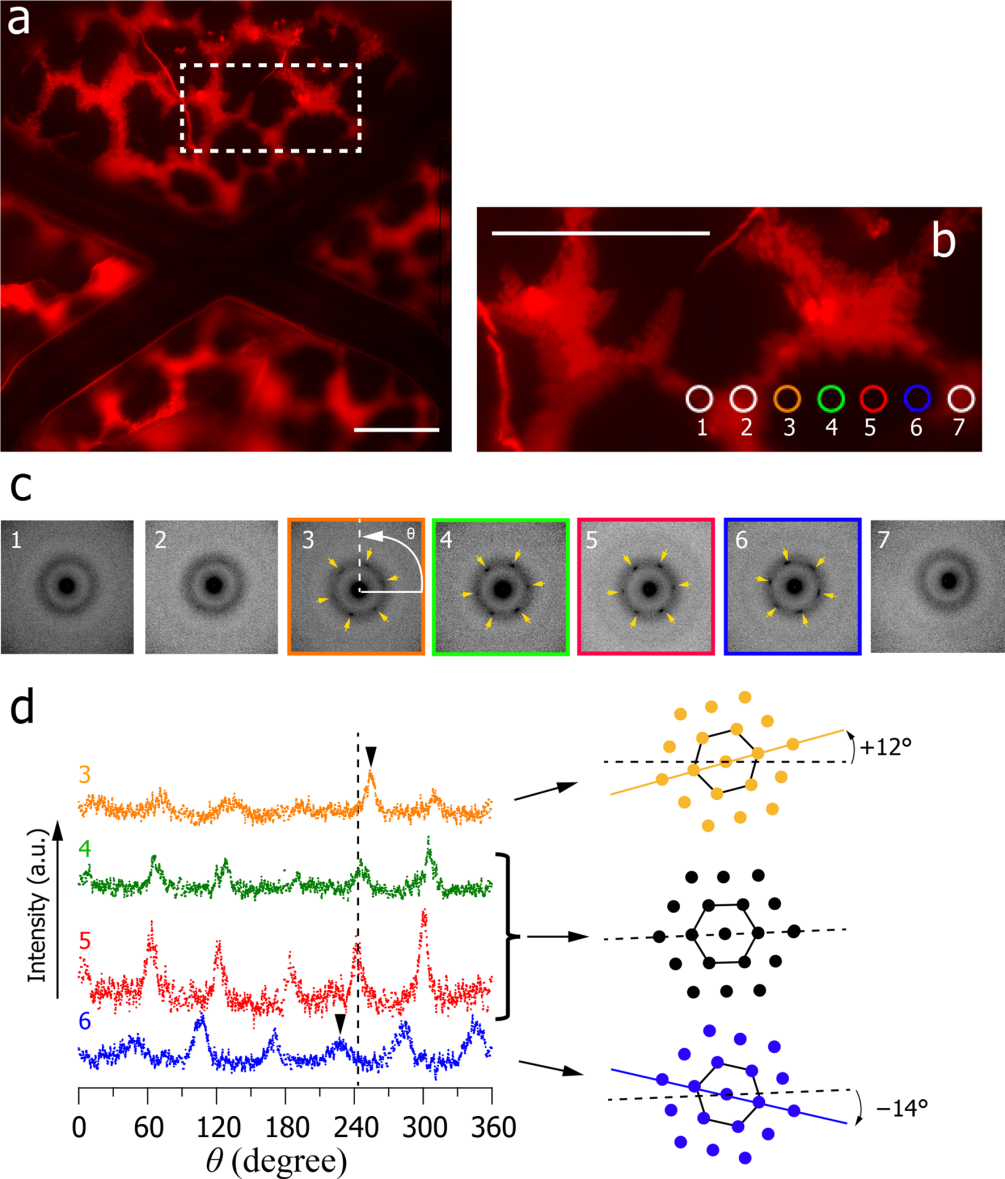
Local structure inside an ordered domain. (**a**) A fluorescent micrograph of the DSPC/DOPC (*x*_DSPC_ = 0.3) monolayer formed on a collodion-coated TEM grid. The darker and brighter regions correspond to the DSPC-rich ordered and DOPC-rich disordered domains, respectively. (**b**) Magnification of the area, which is indicated by the dashed square in (**a**). Bars indicate 30 μm. (**c**) The ED patterns obtained at regions 1–7, as indicated in (**b**). The selected area is 6.2 μm^2^, corresponding to 2.8 μm in diameter. The corresponding region numbers were directly indicated in the panels. (**d**) We plotted the intensity of the hexagonal spots appearing along the azimuthal direction *θ*, which is shown in panel (**c**). The corresponding region numbers are directly indicated in the panel. The difference in the azimuthal angle of the hexagonal spots between the centre (regions 4 and 5) and annular (regions 3 and 6) regions in the ordered domain are indicated by a dashed line and arrowheads, respectively. Schematic illustrations of the directions of the chain packing lattice in regions 3–6 are also shown in (**d**).

**Figure 7:**
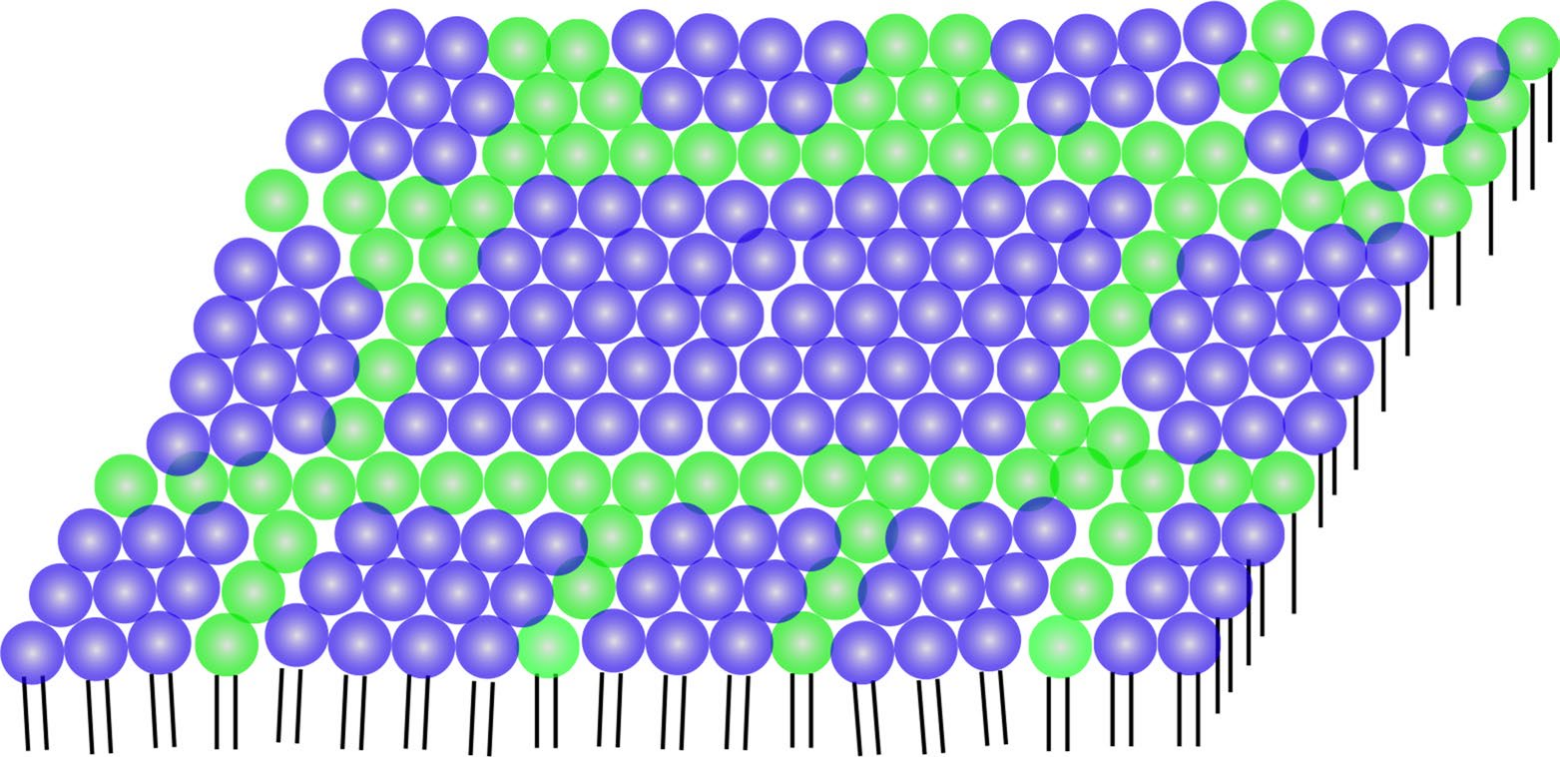
A schematic illustration of the distribution of the subdomains inside a single ordered domain. Blue and green lipids correspond to DSPC and DOPC, respectively.

Finally, taking advantage of the small size of the selected areas in the ED experiments, we compared the chain packing structure at seven different regions across a single ordered domain by low-flux scanning electron diffraction (LFSED). In the LFSED experiment, we reduced the electron flux and exposure time to be 0.8 e/nm^2^·s and 0.5 s, respectively, for further suppression of the beam damage. Then, the size of each region was set to 6.2 μm^2^. As in Fig. 5b, sharp hexagonal spots were obtained in the ordered domain (regions 3–6 in Fig. 6b,c), while a broad Debye–Scherrer pattern was observed in the disordered domain (regions 1, 2, and 7 in Fig. 6b,c). Moreover, the one-dimensional ED pattern showed that the peak positions (2.39 ± 0.02 nm^−1^) are almost the same between regions 3–6 (Supplementary Fig. S4), indicating a similar lattice spacing of the chain packing in the ordered domain. To analyse substructures formed in the ordered domains, we plotted the peak intensity at 2.39 nm^−1^ along the azimuthal direction *θ*. Consequently, it was found that the centre regions of the ordered domain (regions 4 and 5) show diffraction peaks at almost the same angles (dashed line in Fig. 6d). On the other hand, the regions 3 and 6 showed diffraction peaks at slightly different angles from the centre regions: + 12° and − 14°, respectively, (arrowheads in Fig. 6d). These results suggest that a single ordered domain contains multiple subdomains with different crystallographic axes. Here, the regions 4 and 5 belong likely to a same subdomain because it is quite unlikely that two different subdomains are incidentally oriented in the same direction. In such a case, the size of the subdomain is larger at the domain centre than in the vicinity of the phase boundary (Fig. 7).

## Discussion

In the present study, we examined the packing structure of lipid carbon chains inside a single ordered domain using the LFSED and identified that the ordered domain consists of multiple subdomains with different crystallographic axes (Fig. 6d). Moreover, results indicated that the size of the subdomains is larger in the domain centre than in the vicinity of the phase boundary (Figs. 6 and 7). Because the edge of the ordered domain is exposed to a disordered matrix, the packing structure near the phase boundary should be directly affected by inter-domain interactions, such as electrostatic interaction^43^, line tension^44^, and transverse shear when the domains are in motion. It is probable that these interactions hamper the unidirectional arrangement of the chain packing, giving rise to the formation of small subdomains in the vicinity of the phase boundary.

Some previous studies compared the size of the subdomains based on the halfwidth of the WAXD peak^45,46^. However, these studies also suggested that the width of the WAXD peak depends not only on the size of subdomains but also on the other factors such as the order of the chain packing, the instrumental setup, and so on. In that case, the deconvolution of these components is necessary for estimating the size of the subdomain^47^. On the other hand, the LFSED can assess the size of the subdomain without the deconvolution analysis and, thus, this point is remarkable merit for the LFSED when it is compared with WAXD.

Another interesting finding is that the lattice spacing of the carbon chains is almost the same between the pure DSPC monolayer (2.40 ± 0.01 nm^−1^; bottom profiles in Fig. 3a–d) and the DSPC-rich ordered domain (2.39 ± 0.02 nm^−1^; Supplementary Fig. S4), although the *π–A* isotherm measurements showed that the DSPC-rich macro-domain contains small amounts of DOPC (see Fig. 4b and Text). We speculated that, in the DSPC-rich domain, the subdomains are formed by pure DSPC, while small amounts of DOPC molecules reside in the inter-subdomain space (Fig. 7). In this model, since DOPC molecules do not perturb the chain packing of DSPC, the lattice spacing of the DSPC-rich domain should be consistent with that of the pure DSPC monolayer.

Moreover, the pure DSPC monolayer showed a Debye–Scherrer pattern, while the DSPC-rich ordered domain gave the hexagonal spots (compare Figs. 2 and 5b). This means that the size of the subdomain is larger in the DSPC-rich domain than in the pure DSPC monolayer. It is not surprising that even in the pure monolayer, incidentally-created membrane defects often chop off the long-range alignment of carbon chains^48^, leading to the formation of subdomains. Here, the question arises as to why the size of the subdomains is different between the pure DSPC monolayer and the DSPC-rich ordered domain. In the pure system, the van der Waals interaction between the DSPC molecules could be the dominant force for DSPC assembly. On the other hand, in the DSPC-rich domain, molecular contact of the unfavourable pair, DSPC and DOPC, should cause energetic disadvantage^49^. We speculate that, besides the van der Waals interaction, avoidance of unfavourable pairing leads to the formation of a larger subdomain in the DSPC-rich domains. However, to confirm this speculation, we need further study on the correlation between the inter-lipid interactions and the size distribution of the subdomains.

It has been suggested that the substructures inside ordered membrane domains are involved in important biological functions. For example, Wu et al. demonstrated that molecular dynamics in the ordered domains are strongly affected by the substructure and, thus, proposed that the subdomains are responsible for determining the protein partitioning into the ordered domains^17^. In addition, our group reported that a minor structural change of a lipid molecule alters the size of the subdomains, affecting water permeability of the membranes^46^. However, these previous studies could not address the chain packing structure of each subdomain nor the determination of the subdomain size. Therefore, LFSED is promising methodology to understand mechanistic link between the substructure of the ordered domains and their biological functions.

## Conclusion

Electron diffraction (ED) is promising methodology for examining local structures of the lipid membranes due to following reasons. Firstly, ED can assess local structures of lipid membranes. Secondly, due to great diffraction potency of the electron beam, ED can produce strong diffraction peaks even from a single membrane domain. Lastly, ED is easily performed with a usual transmission electron microscope. Compared with WAXD, these points are striking advantages of ED. However, some technical difficulties hamper application of the ED to lipid membranes. For example, the ED experiment requires a dry and fixed sample. In addition, the electron beam causes significant damage to the lipid membranes.

In the present study, we first prepared dehydrated samples using the RFS method and demonstrated that it provides dehydrated monolayer samples without significant shrinkage of the chain-packing lattice in the monolayer samples. We next optimised the intensity of the electron beam to minimise the beam damage to the membrane sample. As a result, we found that the beam damage is sufficiently small to be considered negligible under the electron flux and irradiation durations of less than 4.7 e/nm^2^·s and 5 s, respectively. Finally, we examined the chain packing structure inside the ordered domain, which is formed in the DSPC/DOPC (*x*_DSPC_ = 0.3) phase separated monolayer. Consequently, our LFSED measurement indicated that the ordered domain consists of multiple subdomains with different crystallographic axes (Fig. 6d). Moreover, it is indicated that the size of the subdomain is larger in the domain centre than in the vicinity of the phase boundary (Fig. 7). To the best of our knowledge, this is the first example to address the chain packing structures inside a single ordered domain.

## Materials and methods

### Materials

1, 2-distearoyl-*sn*-3-phosphatidylcholine (DSPC) and 1, 2-dioleoyl-*sn*-3-phosphatidylcholine (DOPC) were purchased form Avanti Polar Lipid Inc. (Alabaster, AL). Fluorescent lipid analogues, 2-(4,4-difluoro-5,7-dimethyl-4-bora-3a,4a-diaza-s-indacene-3-pentanoyl)-1-hexadecanoyl-sn-glyceo-3-phosphocholine (Bodipy-PC) and N-(Texas Red sulfonyl)-1,2-dihexadecanoyl-snglycero-3-phosphatidylethanolamine triethylammonium salt (Texas Red-DPPE) were purchased from Molecular Probe (Eugene, OR). These lipids and fluorescent lipid analogues were dissolved in chloroform/methanol (4:1 v/v) (Wako pure chemicals, Osaka, Japan) and stored at − 40 °C until use.

### Surface pressure *vs*. molecular area isotherm (*π–A* isotherm) measurements and preparation of supported monolayers

Monolayers of lipid mixtures were prepared on a computer-controlled Langmuir film balance (USI System, Fukuoka, Japan) calibrated using stearic acid (Sigma-Aldrich, St. Louis, MO). The lipid solution was prepared by dissolving appropriate amount of DSPC and DOPC in chloroform/methanol (4:1 v/v). Then, 30 μL of lipid solution (1 mg/mL) was spread onto the water subphase (100 × 290 mm^2^) with a glass micropipette (Drummond Scientific Company, Pennsylvania, USA). The monolayers were compressed at a rate of 20 mm^2^/s after an initial delay period of 10 min for the evaporation of organic solvents. The subphase and ambient temperatures were controlled at 25.0 ± 0.1 °C and 25 ± 1 °C, respectively. The measurements were repeated three times under the same conditions and average data were shown.

In the preparation of the supported monolayer, the lipid solution in the presence of 0.2 mol% Texas Red-DPPE was spread on the water subphase. Then, the substrate such as a mica plate and a collodion coated TEM-grid (Okenshoji Co., Ltd, Tokyo, Japan) was horizontally dipped into the water subphase, and the lipid sample was compressed to 30 mN/m at the rate of 20 mm^2^/s. After the compression, the monolayer was transferred onto the substrate. Then, distribution of the ordered and disordered phases in the supported monolayers were observed with a fluorescent microscope BZ-X700 (Keyence, Osaka, Japan).

### Rapid freeze and sublimation (RFS) methods

The dehydrated sample for the electron diffraction experiment was prepared by the RFS method, which is derived from the methods employed in freeze-fracture electron microscopy and mass-based imaging^36−38^. Briefly, immediately after the preparation of the supported monolayer, the wet sample was rapidly frozen in slush N_2_. Then, the sample was stored in a vacuum chamber maintained at 6.7 × 10^−2^ Pa, which is much lower than the triple point of water in the pressure vs. temperature phase diagram. The temperature was gradually increased from – 180 to 25 °C to completely sublimate water molecules from the sample.

### Fluorescent energy transfer (FRET) experiments

Mica supported DOPC monolayers were prepared by aforementioned protocol. In the FRET measurements, the samples contained 0.2 mol% Bodipy-PC and 0.4 mol% Texas Red-DPPE as FRET donor and acceptor, respectively. Then, the samples were dehydrated by the RFS methods or dehydrated at the atmospheric pressure and the room temperature. Then, the intensity of the FRET-donor *I*_donor_ was measured at more than thirty different locations (50 × 50 μm^2^ for each) in the sample with fluorescent microscope BZ-X700 and the average value was shown. The *I*_donor_-value of the wet sample was measured immediately after the preparation of the supported monolayer. The excitation wavelength of 470 nm was applied to Bodipy-PC and the emission were detected at 525 nm using dichroic mirrors OP-87763 (Keyence, Osaka, Japan).

### Electron diffraction (ED) experiments

The local-structure analysis inside a single membrane domain was conducted with transmission electron microscope JEM1400 (JEOL, Tokyo, Japan), and other ED experiments were conducted with JEM2100HCKM (JEOL Ltd., Tokyo, Japan). The supported monolayer mounted on a cooling folder, such as Gatan 626 and Gatan 636 for JEM1400 and JEM2100HCKM, respectively, was set into an electron microscope. The sample was kept at − 180 °C, and the electron beam was irradiated perpendicular to the supported monolayer surface. The instrumental camera length was calibrated using gold particles. Electron diffraction experiments were performed at an accelerating voltage of 100 kV unless otherwise mentioned. The other conditions such as electron flux and exposure time were described in the text. Diffraction patterns were acquired using a CCD camera ES500W (Gatan Inc., Pleasanton, CA) and TemCam-F416 (TVIPS, Gauting, Germany) for JEM1400 and JEM2100HCKM, respectively. To improve visibility of the diffraction patterns, we showed black-and-white inverse images. The value of the electron flux was estimated from the brightness of the digitised image of the incident beam in the absence of a lipid sample (blank image) because the electron flux was too low to be detected by the equipped current meter^35^. Specifically, in the LFSED measurements, the electron flux was calculated by interpolating the brightness of the blank image at 0 pA and 1 pA. Before all experiments, we took a warming-up duration for more than two hours to supply stable electron flux. Referring to the fluorescent micrograph, we applied an electron beam to the target together with acquisition of the diffraction pattern. After the experiment, we determined the beam position using the debris and incidental breaks on the collodion film as landmarks (Supplementary Fig. S3). The diffraction patterns were linearised using Fit 2D and one-dimensional profiles as a function of *s* = 1/d = 2sin*θ*/*λ* were obtained. Here, *s* is modulus of scattering vector, *d* is real spacing of the packing lattice, *θ* is the scattering angle and *λ* is the wavelength of the electron beam. The diffraction peak of the collodion film was subtracted from all the data using a blank sample, which is monolayer-free TEM-grid. The peak position and intensity were estimated by fitting the diffraction profile to the Lorentz function with Origin 2015 (Lightstone, Tokyo, Japan).

According to previous literature^24^, the lateral occupied area of a lipid molecule (*A*_lipid_) is estimated by

1 $$A_{lipid} = \frac{{2 \times A_{chain} }}{\cos \theta }.$$

Here, *A*_chain_ is the lateral occupied area of a carbon chain, and *θ* is the tilt angle of the carbon chain to the membrane normal. If the tilt angle of carbon chains is almost zero, the lateral occupied area of the lipid is easily estimated by

2 $$A_{lipid} = \frac{4}{\sqrt 3 }d^{2} .$$

## Supplementary Information

Supplementary Figures.
