# Supplementary material for: Low-flux scanning electron diffraction reveals substructures inside the ordered membrane domain
Source: Sci Rep. 2020 Dec 21;10:22188. doi: 10.1038/s41598-020-79083-7 (PMC7752913; doi:10.1038/s41598-020-79083-7)
Supplement: Supplementary file 1 — Supplementary Figures. [file 41598_2020_79083_MOESM1_ESM.docx]

**Supplementary Information**

**Low-flux scanning electron diffraction reveals substructures inside the ordered membrane domain**

Masanao Kinoshita*, Shimpei Yamaguchi, Nobuaki Matsumori

Department of Chemistry, Graduate School of Science, Kyushu University, Japan

***Corresponding Authors**

Masanao Kinoshita, Ph D. Sc.

Assistant Professor

Department of Chemistry, Graduate of Science, Kyushu University

Fukuoka 819-0395, Japan

Ph: +81-92-802-4151

Fax: +81-92-802-4148

E-mail: kinoshi@chem.kyushu-univ.jp

Supplementary Fig S1. One-dimensional ED profiles of the DSPC monolayer under an electron flux of 30 e/nm^2^·s. This profile was obtained from the ED pattern shown in Fig. 2e.

Supplementary Figure S2. Kinetics of structural decay upon continuous irradiation of electron beam with a wavelength of 0.0021 nm (*V*_cc_=200 keV). Peak heights are plotted as a function of *t*_irr_ under the electron flux of 4.8 e/nm^2^·s (red plots) and 8.6 e/nm^2^·s (black plots). The dashed lines indicate linear fitting in the region of *t*_irr_ < 77 s for 4.8 e/nm^2^·s and *t*_irr_ < 61 s for 8.6 e/nm^2^·s.

Supplementary Figure S3. Determination of the electron beam position on the DSPC/DOPC (*x*_DSPC_=0.3) monolayers. First, we matched the magnifications between the fluorescent and electron micrographs, referring to the size of the TEM-grid frame. Then, we determined the electron beam position using the debris and incidental breaks formed on the collodion film (indicated by arrows). A bar indicates 30 μm.

Supplementary Figure S4. One-dimensional ED profiles of regions 3–7 shown in Fig. 6b. These profiles were obtained from the ED patterns shown in Fig. 6c. The corresponding region numbers are directly indicated in the figure.
